# Increased IgG4 responses to multiple food and animal antigens indicate a polyclonal expansion and differentiation of pre-existing B cells in IgG4-related disease

**DOI:** 10.1136/annrheumdis-2014-206405

**Published:** 2015-02-02

**Authors:** Emma L Culver, Ellen Vermeulen, Mateusz Makuch, Astrid van Leeuwen, Ross Sadler, Tamsin Cargill, Paul Klenerman, Rob C Aalberse, S Marieke van Ham, Eleanor Barnes, Theo Rispens

**Affiliations:** 1 Nuffield Department of Medicine, Oxford University, Oxford, UK; 2 Translational Gastroenterology Unit, John Radcliffe Hospital, Oxford, UK; 3 Sanquin Blood Supply, Division Research and Landsteiner Laboratory, Academic Medical Center, University of Amsterdam, Amsterdam, The Netherlands; 4 Department of Immunology, Churchill Hospital, Oxford University Hospitals NHS Trust, Oxford, UK; 5 Oxford NIHR BRC, Oxford University, Oxford, UK

**Keywords:** B cells, Inflammation, Autoimmunity, Corticosteroids

## Abstract

**Background:**

IgG4-related disease (IgG4-RD) is a systemic fibroinflammatory condition, characterised by an elevated serum IgG4 concentration and abundant IgG4-positive plasma cells in the involved organs. An important question is whether the elevated IgG4 response is causal or a reflection of immune-regulatory mechanisms of the disease.

**Objectives:**

To investigate if the IgG4 response in IgG4-RD represents a generalised polyclonal amplification by examining the response to common environmental antigens.

**Methods:**

Serum from 24 patients with IgG4-RD (14 treatment-naive, 10 treatment-experienced), 9 patients with primary sclerosing cholangitis and an elevated serum IgG4 (PSC-high IgG4), and 18 healthy controls were tested against egg white and yolk, milk, banana, cat, peanut, rice and wheat antigens by radioimmunoassay.

**Results:**

We demonstrated an elevated polyclonal IgG4 response to multiple antigens in patients with IgG4-RD and in PSC-high IgG4, compared with healthy controls. There was a strong correlation between serum IgG4 and antigen-specific responses. Responses to antigens were higher in treatment-naive compared with treatment-experienced patients with IgG4-RD. Serum electrophoresis and immunofixation demonstrated polyclonality.

**Conclusions:**

This is the first study to show enhanced levels of polyclonal IgG4 to multiple antigens in IgG4-RD. This supports that elevated IgG4 levels reflect an aberrant immunological regulation of the overall IgG4 response, but does not exclude that causality of disease could be antigen-driven.

## Introduction

IgG4-related disease (IgG4-RD) is a multisystem fibroinflammatory condition, characterised by the development of mass lesions with similar histopathological findings in the involved organs.^1^ Histological characteristics include an infiltrate of lymphocytes and plasma cells, a storiform pattern of fibrosis, obliterative phlebitis and variable presence of eosinophils. An elevated serum IgG4 and abundant accumulation of IgG4-positive plasma cells in affected tissues is frequently seen. An increase in circulating plasmablasts and IgG4+ B cells have also been demonstrated.^2^ ^3^ Autoimmune pancreatitis (AIP) and IgG4-related cholangitis (IRC) were the first described manifestations of the disease.^4^

An immune-mediated pathogenesis has been suggested in IgG4-RD, supported by a human leucocyte antigen type II association, presence of autoantibodies and elevated levels of IgG4 and a dramatic response to corticosteroid therapy.^5^ ^6^ Antibodies against a range of autoantigens have been proposed including antinuclear antigens, lactoferrin, carbonic anhydrase II and IV, pancreatic secretory inhibitor and trypsinogens.^7^ ^8^ However, none has been consistently found in the disease, and the fact that they are of the IgG1 and not IgG4 subclass makes their overall significance unclear. A role for *Helicobacter pylori* plasminogen-binding peptide, through a process of antibody cross-reactivity with ubiquitin-protein ligase E3 component n-recognin 2 (molecular mimicry) in genetically predisposed individuals, has been suggested in AIP.^9^ Furthermore, next-generation sequencing of whole blood in patients with IRC highlighted highly abundant IgG4-positive clones in the B cell repertoire, suggesting that specific B cell responses are pivotal to disease pathogenesis.^10^

Our alternative hypothesis is that the elevated IgG4 may not be (primarily) triggered by specific (auto)-antigens, but be an indirect consequence of the expansion of pre-existing IgG4-switched B cells as being responsible for IgG4-RD. In this case, one would expect to find a more generalised and (compared with an antibody response derived from long-lived, bone-marrow-resident plasma cells) a more transient increase in IgG4 antibodies against different antigens that are known to elicit an IgG4 antibody response in the general population. We tested this hypothesis by investigating the level and persistence of the IgG4 response to a variety of known IgG4-inducing non-infectious environmental antigens. We analysed patients with IgG4-RD, patients with primary sclerosing cholangitis (PSC) and elevated IgG4 (a subset of patients with PSC who have an elevated serum IgG4 level but no histological or radiological evidence of IgG4-RD), and healthy controls in a UK cohort.

## Methods

Detailed description of patient inclusion criteria and methodology can be found in the online supplementary repository. Antigen-specific IgG4 responses to egg, milk, peanut, banana, rice, wheat and cat were quantified using a previously developed radioimmunoassay.^11^

## Results

### Characteristics of the cohort

Demographics, clinical characteristics and serum immunoglobulin values of patients and controls are shown in the online supplementary table S1. Most patients with IgG4-RD (83%) had pancreatic (AIP) and/or biliary (IRC) involvement, with 71% having other systemic organ involvement. The concentration of serum total IgG, IgG4, IgE and, to a lesser extent IgG1, was higher in patients with IgG4-RD versus healthy controls, as well as in the patients with PSC-high IgG4 versus healthy controls (see online supplementary figure S1).

### Antigen-specific responses

**Figure 1:**
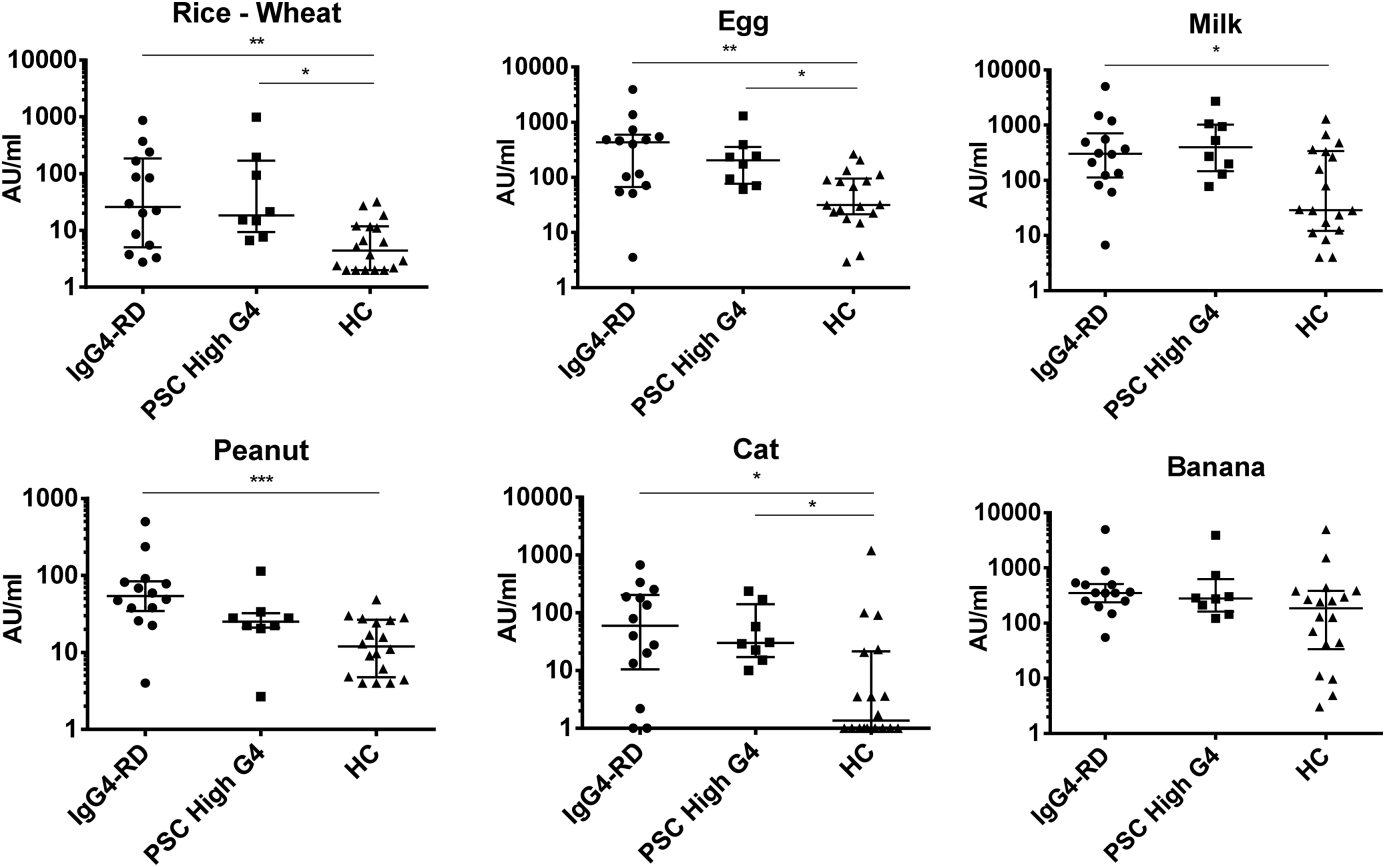
The dot plots show IgG4 antigen-specific responses in treatment-naive patients with IgG4-RD, patients with PSC-high IgG4, and healthy controls. X-axis labels as shown in the figure . Antigens were rice and wheat, egg, milk, peanut, cat dander and serum, and banana. On the Y-axis is IgG4-specific antigen response, log 10 scale, in arbitrary units per mL. Error bars represent median and IQR; p values: *p<0.05, **p<0.005, ***p<0.001. HC, healthy controls; IgG4-RD, IgG4-related disease; PSC, primary sclerosing cholangitis.

We analysed the IgG4 response to proteins from egg, milk, peanut, banana, rice, wheat and cat. In line with our hypothesis, the response to egg (p=0.004), milk (p=0.04), peanut (p=0.0003), cat dander and serum (p=0.012), rice and wheat (0.006) antigens was found to be higher in patients with IgG4-RD than in healthy controls, and the response to egg (p=0.03), cat dander and serum (p=0.04), and rice and wheat (p=0.01) antigens was higher in patients with PSC-high IgG4 than in healthy controls (figure 1).

We further examined the correlation of serum immunoglobulin levels with antigen-specific responses. In patients with IgG4-RD, there was a positive correlation between serum IgG4 levels and IgG4 responses to banana (Rank 0.38, 95% CI −0.002 to 0.67, p=0.045), peanut (Rank 0.49, 95% CI 0.14 to 0.74, p=0.007), cat (Rank 0.61, 95% CI 0.29 to 0.80, p=0.0006), rice and wheat (Rank 0.38, 95% CI −0.002 to 0.67, p=0.045) antigens (see online supplementary figure S2). By contrast, total serum IgE levels did not correlate with antigen-specific responses (data not shown).

### Treatment-naive and treatment-experienced patients with IgG4-RD

**Figure 2:**
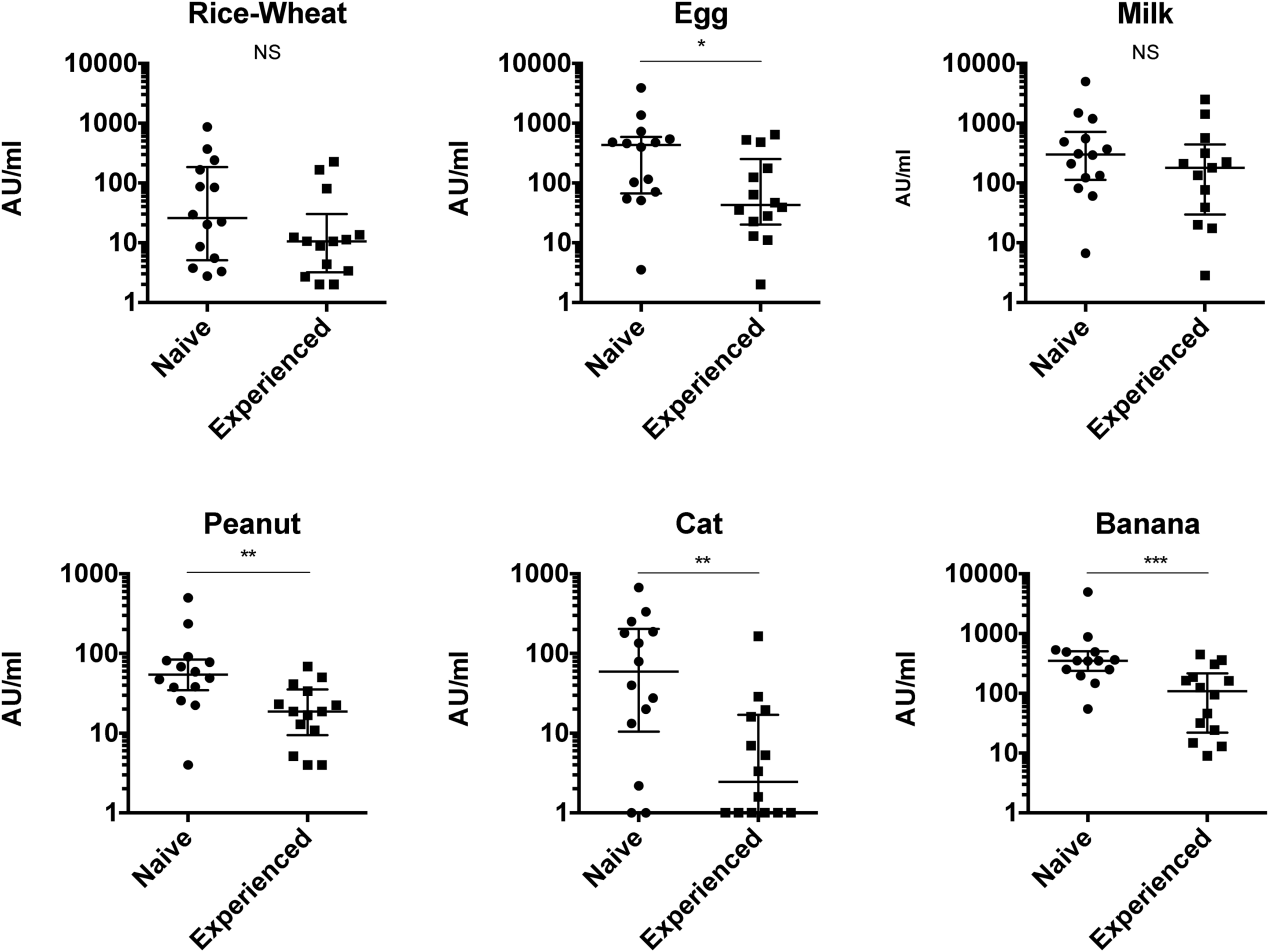
The dot plots show the IgG4 antigen-specific responses in treatment-naive and treatment-experienced (on corticosteroids) patients with IgG4-related disease (IgG4-RD). Antigens were rice and wheat, egg, milk, peanut, cat dander and serum, and banana. Units as in figure 1. Error bars represent median and IQR. Mann–Whitney p values: *p<0.05, **p<0.005, ***p<0.001.

We also tested the differences in immunoglobulin levels and IgG4-specific antigen responses in 14 treatment-naive and 10 treatment-experienced patients with IgG4-RD receiving corticosteroid therapy. Levels of antibodies to banana (p=0.001), egg (p=0.039), peanut (p=0.003) and cat (p=0.006) antigens were lower in treatment-experienced compared with treatment-naive patients (figure 2), as were serum total IgG (p=0.017) and IgG4 (p=0.001) (see online supplementary figure S3).

### Serum electrophoresis

We analysed serum electrophoresis data for evidence of monoclonality in patients and controls, but no monoclonal bands in the polyclonal gamma region were observed (see online supplementary figure S4). All individuals showed polyclonality; 11/14 (78.6%) patients with IgG4-RD, 3/9 (33.3%) PSC-high patients with IgG4, and 0/18 healthy controls showed hypergammaglobulinaemia. Further immunofixation analysis of patient samples with a diffuse increase in immunoglobulins, using IgG, IgA, IgM, kappa and lambda light-chain antisera, confirmed no monoclonal bands in the gamma region (see online supplementary figure S4).

## Discussion

Our data show that IgG4-RD is associated with an elevated IgG4 response to diverse antigens. Therefore, elevated levels of IgG4 in patients with IgG4-RD may be the result primarily of a polyclonal expansion of many IgG4 B cells irrespective of their specificity. This would imply that factors other than antigen induce an expansion and differentiation of IgG4 B cells, but does not exclude the possibility that a single antigen (self or exogenous) serves as the initial trigger of disease. Antigen-independent involvement of many IgG4-switched B cells implies differential selection via signals that uniquely act on IgG4 B cells. We have recently demonstrated differences in phenotype between IgG1 and IgG4 B cells,^12^ and future studies may reveal traits that uniquely link IgG4 B cells to proliferation in inflamed or fibrotic tissue.^13^ The IgG4-switched B cells may be selectively triggered to expand and differentiate into plasma cells. This may involve interleukin (IL)-21 driving proliferation and expansion of IgG4-switched cells and the upregulation of activation-induced cytidine deaminase, B-lymphocyte-induced maturation protein 1 and Xbox protein 1, all of which have been shown to be upregulated in patients with various organ manifestations of IgG4-RD.^14^ ^15^ Alternatively, signals from the inflamed/fibrotic tissue, including cytokines such as IL-4/IL-13 or IL-10 could selectively stimulate B cells to proliferate into IgG4 plasma cells, either directly or via Th2 and T regulatory cells.^16^ ^17^ Such an environment may be actively sustained via signals from IgG4 B cells themselves, possibly involving IL10.^3^ ^18^ Furthermore, since IgG4 responses are associated with chronic stimulation, this B cell subset may be more susceptible to expansion due to continued exposure to antigen(s), and therefore there does not need to be a functional link between antibody specificity and potential autoantigen. Importantly, these scenarios are not mutually exclusive. However, it remains unclear if the elevated IgG4 reflects a causal event or epiphenomena. Further research is necessary to determine if the large amounts of IgG4 plasma cells are predominantly the result of disease-associated class switch or disease-associated expansion and terminal differentiation of IgG4 B cells.

The fall in IgG4-specific antigen responses after corticosteroids may be explained by suppressed proliferation of expanded IgG4-switched B cells or suppressed differentiation into IgG4 plasmablasts. Corticosteroids interfere with the production of cytokines critical for T cell proliferation and interaction, and the binding of interleukins to B cells suppressing proliferation and antibody production. The steroid effect highlights the rapid turnover of the IgG4-producing plasma cells, as opposed to production by long-lived plasma cells in the bone marrow.

Evidence for polyclonality in IgG4-RD is supported by the diversity of antigen specificity, and by serum electrophoresis and immunofixation analysis no monoclonal bands in the polyclonal gamma region was observed (see online supplementary figure S4). However, this is complicated by the ability of the bispecific IgG4 antibody to undergo Fab-arm exchange (known to take place in vivo^12^), which limits the potential to detect an oligoclonal response where multiple clones have comparable abundance. In model systems using chimeric antibodies, Fab arm exchange can affect the electrophoretic mobility of IgG4, although if >50% of the IgG4 would be monoclonal then >25% would still remain monoclonal after Fab-arm exchange. Recently, oligoclonal somatically hypermutated plasmablast populations were demonstrated by next-generation sequencing in patients with active IgG4-RD.^19^

Mechanisms responsible for driving IgG4-RD (eg, Th2 cytokines) may also increase IgE B cell expansion in certain individuals. In this instance, one may expect an increased predisposition to allergies in later years of life. However, the frequency of allergy/atopy was similar in patient and control groups (see online supplementary table S1). Furthermore, there was no significant difference between total and antigen-specific IgG4 responses in patient and control groups (not shown). However, serum IgE levels in patients with IgG4-RD with allergy/atopy was higher than in those without (p=0.0255) (see online supplementary figure S3); consistent with Della Torre *et al*^20^ Therefore, it is plausible that elevated serum IgG4, resulting from polyclonal expansion of many IgG4 B cells irrespective of antigen specificity, may be linked to IgE B cell expansion in a subset of allergic/atopic individuals with IgG4-RD.

To summarise, this is the first study to show an enhanced polyclonal IgG4 response to multiple non-infectious environmental antigens in IgG4-RD. Multiple different antigen responses are higher in treatment-naive compared with treatment-experienced patients. The elevated IgG4 levels may reflect the aberrant immunological regulation of the overall IgG4 response in the disease.

## Supplementary Material

Web supplement

Web table
