# Supplementary material for: Increased IgG4 responses to multiple food and animal antigens indicate a polyclonal expansion and differentiation of pre-existing B cells in IgG4-related disease
Source: Ann Rheum Dis. 2015 Feb 2;74(5):944–7. doi: 10.1136/annrheumdis-2014-206405 (PMC4392210; doi:10.1136/annrheumdis-2014-206405)

**Supplementary Data**

**Methods**

**Patient and control cohorts: definitions and recruitment**

Patients and controls were recruited from the John Radcliffe Hospital in Oxford, a tertiary referral centre for IgG4-RD and PSC in the UK. Patients with IgG4-RD who were referred to Oxford from February 2005 to February 2013 and followed prospectively were recruited. Diagnosis of IgG4-RD was made using the HISORt criteria (since 2007), and the International Consensus Diagnostic Criteria (ICDC) (since 2011),[1, S1]. The Boston Consensus Histopathological Criteria for IgG4-RD (2012) was applied to those with biopsy and surgical resection specimens,[S2]. Patients with primary sclerosing cholangitis (PSC) referred to Oxford from January 2002 to February 2013 and followed prospectively were recruited. Diagnosis of PSC was made in accordance with the European Association for the Study of the Liver Guidelines in those with a cholestatic biochemical profile, characteristic cholangiography and/or consistent liver histology when secondary sclerosing cholangitis had been excluded,[S3]. Healthy controls (HC) with no history of autoimmune conditions were recruited. The European Academy of Allergy and Clinical Immunology classification was used to record a history of allergy or atopy,[S4]. All patients gave written informed consent. Ethical approval was obtained from the Research Ethics Committee, Oxfordshire (ref: 10/H0604/51).

**Serum immunoglobulin measurements**

Serum levels of IgG and subclasses (IgG1 and IgG4) were measured by nephelometry using a Siemens BNII analyser (Siemens, Surry, UK).  Total IgE was measured by the Immunocap method using a Phadia 250 (Phadia, Milton Keynes, UK). Both sets of assays were carried out at the Department of Immunology, Churchill Hospital OUH Trust, Oxford, UK. The

normal range for serum immunoglobulin concentrations was determined by the institute reference values; IgG 6-16.2g/l, IgG1 3.2-10.2g/l, IgG2 1.2-6.6g/l, IgG3 0.2-1.9g/l, IgG4 0.1-1.3g/l and IgE 5-120KU/L. For this study, an elevated serum IgG4 was defined as ≥1.4g/l as used in the Mayo HISORt criteria for AIP[3] and an elevated IgE as >120KU/L as per institute reference. For IgE units are expressed as KU/L, equivalent to 1 IU/mL, and equivalent to 2.4ng/mL. Serum values for IgG subclasses were comparable at Oxford and Sanquin institutes. The prozone effect was avoided by testing samples at serial dilutions.

**Inclusion criteria for the study**

A total of 60 patients with IgG4-RD met diagnostic criteria, of whom 53 patients (88.3%) had an elevated serum IgG4. Serum from 24 patients (23 with elevated serum IgG4 and 1 with normal serum IgG4) was collected at disease presentation for the 14 treatment-naïve patients. For the treatment-experienced patients (n=10), serum was collected at 4-8 weeks after starting a high dose (30-40 mg prednisolone) of corticosteroids. The treatment-experienced group included 4 samples from patients also included in the naive group. No patient was on concomitant immunosuppression. A total of 220 patients met PSC criteria, of whom 33 patients (15%) had an elevated serum IgG4. Serum from 9 PSC patients with elevated serum IgG4 were collected at recruitment, with no concurrent corticosteroid or immunosuppressive medication, imaging evidence to confirm no extra-biliary involvement except inflammatory bowel disease, and no evidence on liver biopsy of IRC morphology. A total 50 healthy controls were recruited, of whom 2 controls (4%) had an elevated serum IgG4. Serum from 18 controls with normal serum IgG4 and no history of autoimmune disease, corticosteroid or immunosuppressive therapies were included.

**IgG4 Radio Immunoassay (RIA)**

The radio immunoassay (RIA) is a sensitive *in vitro* test to determine and quantify antigen-specific antibodies in serum. For the RIA, 250µl of a 2 mg/ml Sepharose suspension (antigen coupled to CNBr-activated Sepharose-4B (Amersham Pharmacia Biotech AB)) was washed (x3) then incubated with 50µl of 1:50 diluted patient or control serum plus 450µl of PBS with human serum albumin, Tween-20 (with mannoside in the case of banana to avoid measurement of non-specific IgG4 due to the presence of lectins in banana), overnight at RT,[9, S5]. The suspension was washed (x5) with PBS containing 0.005% Tween-20 (PBS-T) to remove unbound IgG4 antibodies and other irrelevant serum components, then 500µl of PBS with 1mg/ml sheep IgG and 3mg/ml human serum albumin was added to 50µl of monoclonal mouse-anti-human IgG4 (radiolabelled $^{125}$I (Sanquin)) and left overnight at RT. After washing 5 times with PBS-T, bound radioactivity was measured. Bound radioactivity was converted to arbitrary units (AU) using a calibrator serum with IgG4 to grass pollen set at 1400 AU/ml; one AU is estimated to equal approximately 50 ng IgG4.

The antigens used were egg white and yolk, milk, cat dander and serum, banana, milk, rice and wheat (Sanquin Institute, Amsterdam). All foods were obtained locally, cat dander from HAL (Haarlem, The Netherlands), and cat serum from a local veterinarian. Antigen extracts (for example, egg white and yolk) were separately coupled to beads, but the beads were combined before testing with serum. The amounts of antigen extracts coupled to dry weight Sepharose are: egg white extract 200 mg coupled to 40 g Sepharose-4B; egg yolk extract 160 mg coupled to 20g Sepharose-4B; cat dander extract 100 mg coupled to 20g Sepharose-4B; cat serum ~~extract~~ 5.5 mg coupled to 0.8g Sepharose-4B; banana extract 81 mg coupled to 2g Sepharose-4B; milk extract 609 mg coupled to 10g Sepharose-4B; rice and wheat extracts 240 mg coupled to 6 g of Sepharose-4B. To compare results, signals were compared to a standard curve (a serially diluted reference serum incubated with 4mg/ml Dactylis Sepharose)

and expressed as arbitrary units. Mixtures of chimeric antibodies (anti-Bet v1 and anti-Feld 1) were used to exclude the risk of second-generation IgG4 binding.

**Serum Electrophoresis and Immunofixation**

Serum electrophoresis was performed to check for evidence of monoclonality. This was carried out using a Sebia hydrasys focusing system (Sebia Cedex, France). Initial gel electrophoresis of samples was performed using the Sebia Hydragel 54 protein gel kit (Sebia, Cedex, France). Densitometry of electrophoresis gels was analysed using Phoresis (Sebia, Cedex, France). In patients where no monoclonality was detected, individuals were designated as hypogammaglobulinaemic, normal or hypergammaglobulinaemic, determined by the levels of serum IgG and the appearance of the beta/gamma region of densitometry plot. In samples with a diffuse increase in immunoglobulins, immunofixation analysis using IgG, IgA, IgM, kappa light chain and lambda light chain antisera was carried out (Sebia hydragel IF 2/4 kit (Sebia, Cedex, France)) as a higher sensitivity method to test for the presence of monoclonal bands in the polyclonal gamma region. This method has a lower limit of detection of approximately 0.2g/l of protein.

**Statistical analysis**

A Kruskal-Wallis test with multiple comparisons and an ANOVA using log-transformed data were used to compare IgG4-specific antigen responses in IgG4-RD patients, PSC patients and healthy controls. A two-tailed Mann Whitney was used to compare responses in treatment-naïve and treatment-experienced IgG4-RD patients. Statistics were calculated using Graphpad Prism v6.0. A P-value of $<0.05$ was considered statistically significant.

**Supplementary References for methods section**


S1 Shimosegawa T, Chari ST, Frulloni L, Kamisawa T, Kawa S, Mino-Kenudson M, *et al*. International Consensus Diagnostic Criteria for Autoimmune Pancreatitis. Pancreas 2011;40:352-8.

S2 Deshpande V, Zen Y, Chan JK, Yi EE, Sato Y, Yoshino T, *et al*. Consensus statement on the pathology of IgG4-related disease. Mod Pathol 2012;25:1181-92.

S3 Beuers U, Boberg KM, Chapman RW, Chazouilleres O, Invernizzi P, Jones DE, et al. EASL Clinical Practice Guidelines: management of cholestatic liver diseases. J Hepatol. 2009 Aug;51(2):237-67.

S4 Johansson SG, Hourihane JO, Bousquet J, Bruijnzeel-Koomen C, Dreborg S, Haahtela T *et al*. A revised nomenclature for allergy. An EAACI position statement from the EAACI nomenclature task force. Allergy 2001;56:813–824.

S5 Koshte VL, Aalberse M, Calkhoven PG, Aalberse RC. The potent IgG4-inducing antigen in banana is a mannose-binding lectin, BanLec-I. Int Arch Allergy Immunol 1992;97(1):17-24.

**Supplementary Figures:**

**Figure S1**: The dot plots show serum IgG, IgG1, IgG4 and IgE levels in treatment-naïve IgG4-RD patients, PSC high IgG4 patients and healthy controls. On the X-axis is IgG4-RD – IgG4-RD patients, PSC High G4 –PSC patients with elevated serum IgG4 levels, HC – healthy controls with normal serum IgG4. On the Y-axis is serum immunoglobulin levels, log 10 scale, IgG, IgG1 and IgG4 in grams per litre and IgE in recognised international units KU/L. Error bars represent median and interquartile range. Kruskal-Wallis test with multiple comparison p values * p<0.05, ** p<0.005, ***p<0.001.

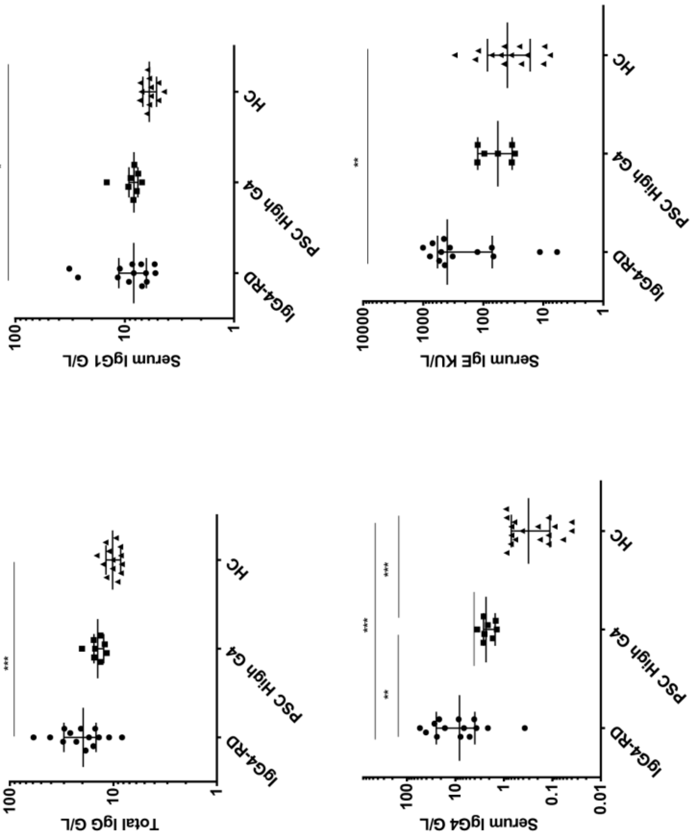

**Figure S2:** Correlation plots of serum IgG4 and IgG4-specific antigen responses in treatment-naïve and experienced IgG4-RD patients. Antigens were rice and wheat, egg, milk, peanut, cat dander and serum, banana.  On the X-axis is serum IgG4, log 10 scale, in grams per litre. On the Y axis is IgG4 specific antigen response, log 10 scale, in arbitary units per ml. Spearman rank correlation R values and p values * p<0.05, ** p<0.005, ***p<0.001.

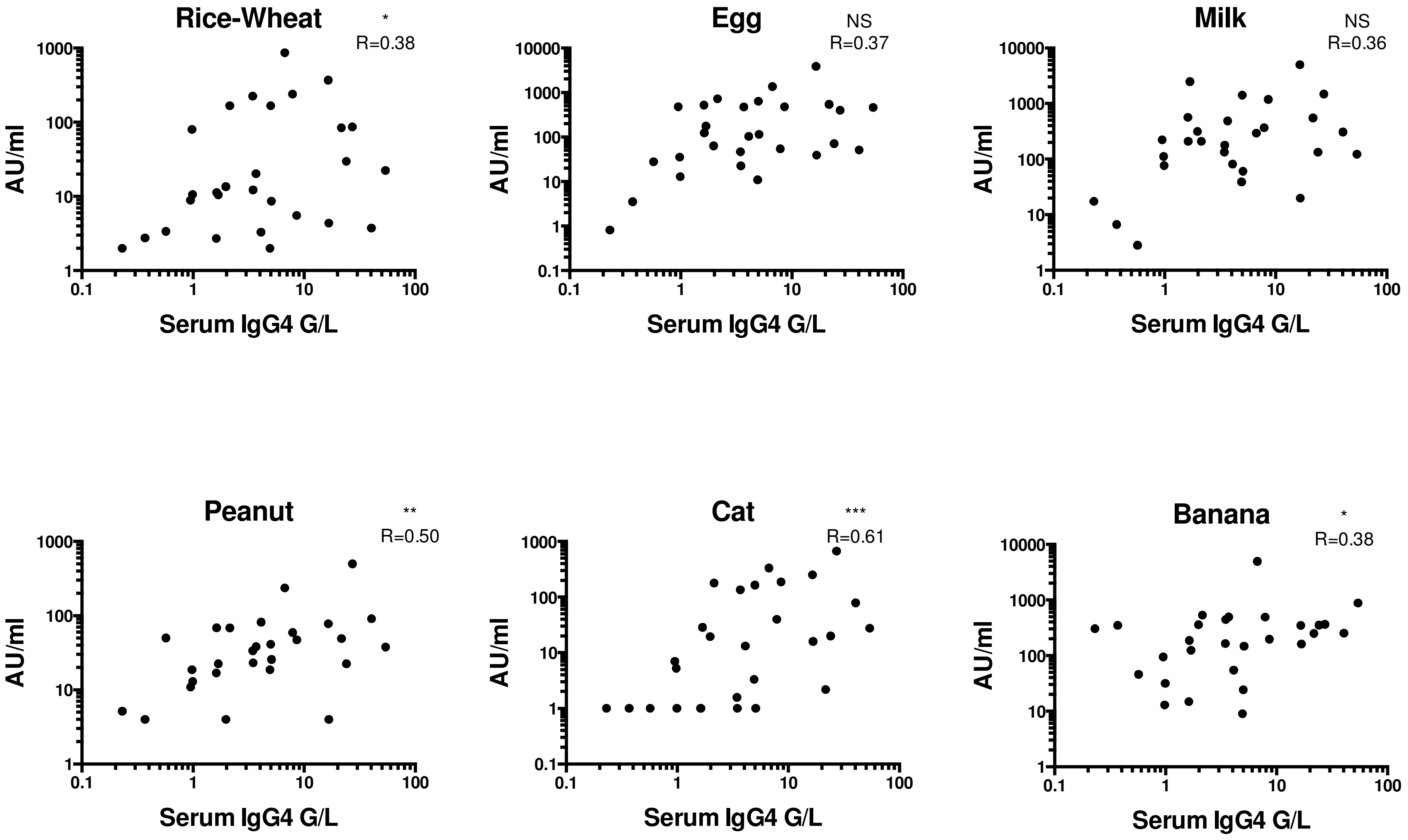

**Figure S3**: The dot plots show the serum IgG, IgG1, IgG4, IgE levels and IgG4/IgE ratio in treatment-naïve and experienced IgG4-RD patients. Units as in Figure S1. Error bars represent median and interquartile range. Lower right panel: IgE levels in IgG4-RD patients with and without atopy. Mann U Whitney p values * p<0.05, ** p<0.005, ***p<0.001.

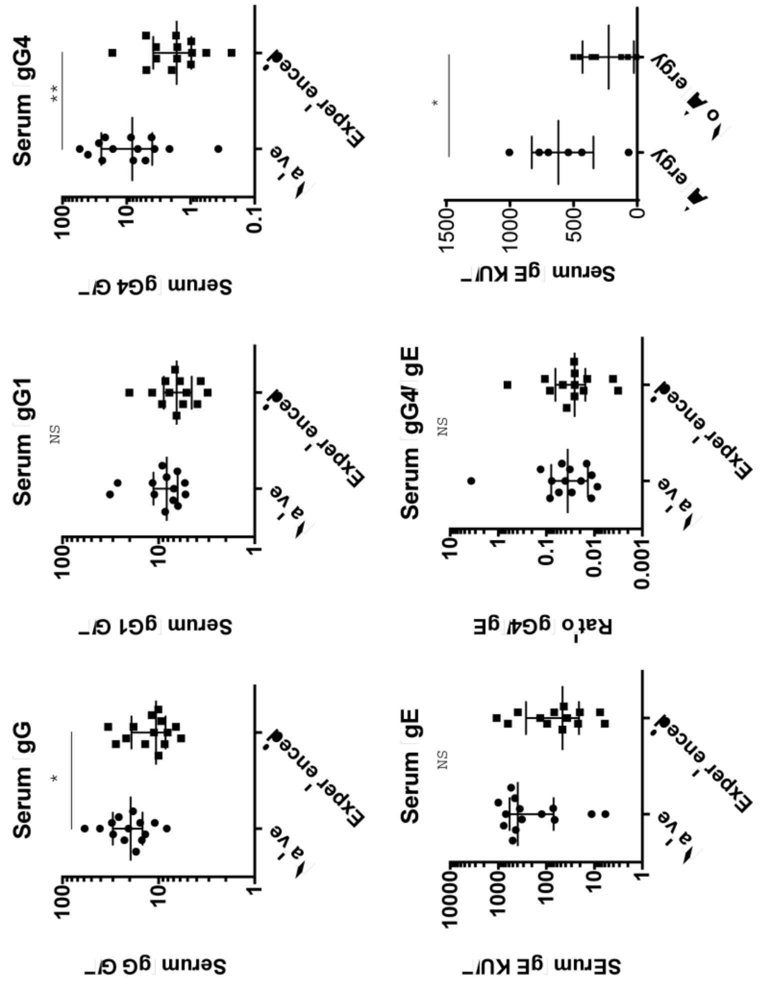

**Figure S4:** A. Representative serum protein electrophoresis gel in a patient with multiple myeloma (M), patient with IgG4-RD (G4) and a healthy control (HC). B. Representative negative serum protein immunofixation using antisera directed at heavy chains of IgG, IgA and IgM plus Kappa (κ) and Lambda (λ) light chain in an IgG4-RD patient who displayed hypergammaglobulinaemia. ELP is the original standard gel control.

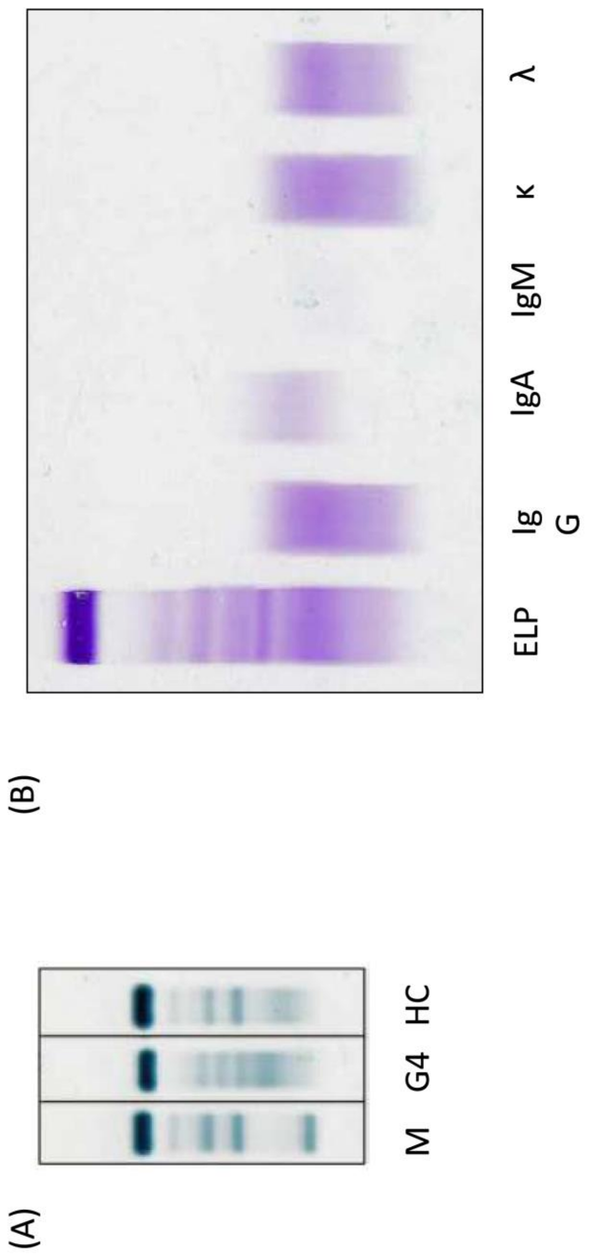

Supplement: Web supplement [file annrheumdis-2014-206405-s1.pdf]
