# Supplementary material for: Increased IgG4 responses to multiple food and animal antigens indicate a polyclonal expansion and differentiation of pre-existing B cells in IgG4-related disease
Source: Ann Rheum Dis. 2015 Feb 2;74(5):944–7. doi: 10.1136/annrheumdis-2014-206405 (PMC4392210; doi:10.1136/annrheumdis-2014-206405)
Supplement: Web table [file annrheumdis-2014-206405-s2.pdf]

## Supplementary Table

**Table S1**: Demographics, characteristics and immunoglobulin values of the cohort.

| | IgG4-RD patients | PSC high IgG4 | Healthy controls | P values (ANOVA) | IgG4-RD vs PSC (KW) | IgG4-RD vs HC (KW) | PSC vs HC (KW) |
|---|---|---|---|---|---|---|---|
| **Absolute number** | 24<br>14 treatment-naïve<br>10 treatment-experienced | 8 | 18 | | | | |
| **Age years median (range)** | 65 (32-84) | 65 (35-76) | 48.5 (24-67) | 0.001 | 0.7085 | 0.0007 | 0.0905 |
| **Gender (male) %** | 87.5 | 62.5 | 55.6 | 0.608 | | | |
| **Organs involved AIP/IRC % Systemic OOI %** | 83.3<br>70.8 | 0<br>0 | 0<br>0 | | | | |
| **Allergy or atopy %** | 41.67 | 25.0 | 37.5 | 0.073 | | | |
| **Serum IgG G/L median (range)** | 19.55 (8.24-59.1) | 14.15 (11.6-20.1) | 10.22 (8.3-14.5) | <0.0001 | 0.0727 | <0.0001 | 0.1054 |
| **Serum IgG1 G/L median (range)** | 8.27 (5.25-32.2) | 8.26 (6.94-14.6) | 5.98 (4.37-7.31) | 0.0104 | 0.9512 | 0.0126 | 0.0622 |
| **Serum IgG4 G/L median (range)** | 8.22 (0.37-54.1) | 2.33 (1.4-3.53) | 0.31 (0.04-0.9) | <0.0001 | 0.0186 | <0.0001 | 0.0002 |
| **Serum IgE G/L median (range)** | 397.5 (5.9-1006.0) | 57.1 (29.6-125) | 39.65 (7.75-300) | 0.0025 | 0.1148 | 0.0019 | 0.5994 |

**Abbreviations**: IgG4-RD ‘IgG4-related disease’; PSC ‘primary sclerosing cholangitis’; AIP ‘autoimmune pancreatitis’; IRC ‘IgG4-related cholangitis’; OOI ‘other organ involvement’; KW ‘Kruskal-Wallis test with multiple comparisons’.
